# Dysregulated signalling pathways in innate immune cells with cystic fibrosis mutations

**DOI:** 10.1007/s00018-020-03540-9

**Published:** 2020-05-04

**Authors:** Samuel Lara-Reyna, Jonathan Holbrook, Heledd H. Jarosz-Griffiths, Daniel Peckham, Michael F. McDermott

**Affiliations:** 1 Leeds Institute of Rheumatic and Musculoskeletal Medicine, University of Leeds, Leeds, LS9 7TF UK; 2 Leeds Institute of Medical Research at St James’s, University of Leeds, Leeds, LS9 7TF UK; 3 Leeds Cystic Fibrosis Trust Strategic Research Centre, University of Leeds, Leeds, LS9 7TF UK; 4 Adult Cystic Fibrosis Unit, St James’s University Hospital, Leeds, LS9 7TF UK

**Keywords:** Cystic fibrosis, Inflammation, Neutrophils, Monocytes, Macrophages, CFTR and autoinflammation

## Abstract

Cystic fibrosis (CF) is one of the most common life-limiting recessive genetic disorders in Caucasians, caused by mutations in the cystic fibrosis transmembrane conductance regulator (CFTR). CF is a multi-organ disease that involves the lungs, pancreas, sweat glands, digestive and reproductive systems and several other tissues. This debilitating condition is associated with recurrent lower respiratory tract bacterial and viral infections, as well as inflammatory complications that may eventually lead to pulmonary failure. Immune cells play a crucial role in protecting the organs against opportunistic infections and also in the regulation of tissue homeostasis. Innate immune cells are generally affected by CFTR mutations in patients with CF, leading to dysregulation of several cellular signalling pathways that are in continuous use by these cells to elicit a proper immune response. There is substantial evidence to show that airway epithelial cells, neutrophils, monocytes and macrophages all contribute to the pathogenesis of CF, underlying the importance of the CFTR in innate immune responses. The goal of this review is to put into context the important role of the CFTR in different innate immune cells and how CFTR dysfunction contributes to the pathogenesis of CF, highlighting several signalling pathways that may be dysregulated in cells with CFTR mutations.

## Introduction: an overview of cystic fibrosis

Cystic fibrosis (CF) is one of the most common life-threatening autosomal recessive genetic diseases in Caucasians, affecting approximately 48,000 individuals in Europe and 30,000 in the USA [1, 2]. This condition is caused by mutations in the cystic fibrosis transmembrane conductance regulator (CFTR), which is a transmembrane ion channel highly expressed by cells of the respiratory, digestive and male urogenital tracts [3–8]. Epithelial and secretory cells are known to be profoundly affected by CFTR mutations, due to altered physiological function as a result of abnormal production, maturation and function of the CFTR protein and aberrations in ion transport, most importantly chloride ions (Cl^−^) [9]. These changes result in a multisystem disease characterised by recurrent pulmonary infections, pancreatic insufficiency, gastrointestinal complications, CF-related diabetes, malnutrition, arthropathies and male infertility [10–17]. Until recently, treatments for CF were based on managing disease symptoms and complications rather than treating the underlying disease. Approximately 1 in 25 people in the UK is an asymptomatic carrier of a mutated CFTR gene and likewise 1 in 29 people in the USA.

Drugs used to treat CF frequently include pancreatic enzymes, mucolytics and, antibiotics, including low dose macrolides and other anti-inflammatory agents [18, 19]. The recent introduction of effective CFTR modulator therapy is revolutionising the management of CF. These drugs have variable efficacy and target the underlying problem by improving CFTR expression and function. Drug efficacy is variable with each drug or drug combination and they target specific CFTR class mutations; the drugs include Ivacaftor (VX-770), Lumacaftor (VX-809), Tezacaftor (VX-661) and Elexacaftor (VX-445). Ivacaftor is used to treat class III gating and residual function mutations (e.g. G551D, S549R and V250F), whereas the combination of Ivacaftor/Lumacaftor (Orkambi) and Ivacaftor/Tezacaftor (Symkevi) is effective for patients homozygous for ∆F508 and those with one copy of ∆F508 as well as another residual function mutation [20, 21]. The recent approval of the triple-drug combination therapy, Ivacaftor/Tezacaftor/Elexacaftor (Trikafta) in the USA, heralds a paradigm shift in the treatment of CF, as this combination is highly efficacious for both patients who are homozygous and heterozygous for the ∆F508 mutation [20, 21]. The different types of class mutations, including the most common examples and the current individual treatments, for each type of class mutation, are summarised in Table 1.

**Table 1:**
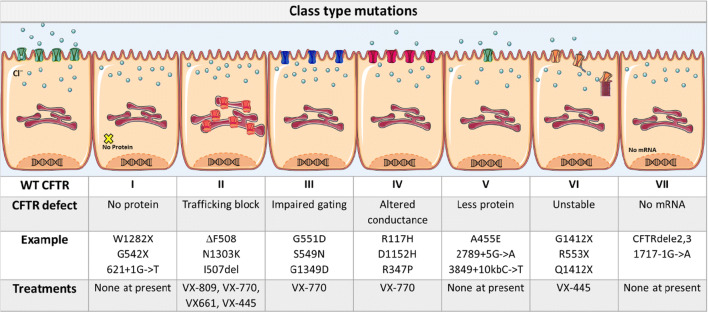
CF class mutations and current therapies

A summary of the seven different classes of CF mutations described above [214, 215]; alongside the most common examples and current treatments

Although initially, just a few studies highlighted the importance of the CFTR in the regulation of immune cell function, there is now more evidence to show the relevance of the CFTR expression in different cells of the immune system [22–30]. Several studies have revealed that specific cellular signalling pathways, of different immune cells with various CFTR mutations, are affected in CF [22–30]. Moreover, CF has been described as an autoinflammatory condition [28, 31], based on the abnormal inflammatory activity of innate immune cells, which is exacerbated by the atypical local tissue environment [27, 28]. Autoinflammatory conditions are primarily driven by aberrant activation of the innate immune system [32, 33], which usually leads to abnormal production of pro-inflammatory cytokines such as TNF, IL-1β, IL-6, IL-17 and IL-18, whereby the local environmental factors may predispose the cells to an inflammatory phenotype [34, 35]. Autoimmune responses, in contrast, are directed against self-antigens and are characterised by the presence of autoreactive T cells and B cell-mediated autoantibodies [34]. Only a limited number of studies have reported the involvement of adaptive immune cells, such as T and B cells, affected by CFTR mutations in CF pathology [36–39]. Therefore, this review will explore existing reports in the literature detailing several mechanistic signalling pathways dysregulated in innate immune cells harbouring CFTR mutations.

## Cellular signalling pathways

The cellular machinery is actively regulated by multiple complex signalling pathways, working together to maintain cellular fitness. Most of these pathways are functionally redundant, as a compensatory mechanism for reduced or absent activity of other similar pathways. For instance, different types of cellular death have been described in mammalian cells, including apoptosis (programmed cell death with chromatin condensation), necrosis (premature cellular death that causes membrane rupture and organelle release), pyroptosis (pro-inflammatory programmed cell death causing cellular destruction) and autophagic cell death (degradation of cellular components in an autophagic manner, leading to cellular death) [40]. All these mechanisms may result in a similar outcome; namely, cellular death; nevertheless, each mechanism accomplishes its primary function via different signalling pathways.

Cysteinyl aspartate-specific proteases, better known as caspases, are the most critical active enzymes involved in the execution of cellular apoptosis and pyroptosis [41, 42]. In terms of apoptotic cellular death, caspases can be divided into two leading families, initiator caspases (− 2, − 8, − 9, − 10) and executioner caspases (− 3, − 6, − 7) [42]. Apoptosis can be induced by extrinsic cellular signals, e.g. TNF, or by intrinsic cytosolic insults, e.g. DNA damage, which eventually lead to cellular death [41, 42]. In contrast, pyroptotic cellular death frequently involves the formation of different inflammasomes, a critical mechanism in the induction of inflammation by innate immune cells [41, 43, 44]. Inflammasomes are cytosolic multiprotein complexes usually consisting of three main domains, the central sensory NOD-like receptor (NLR) domain, the adaptor protein apoptotic speck protein containing a caspase recruitment domain (ASC) domain and pro-caspase-1 domain [44, 45]. Several different inflammasomes have been described in mammalian cells, including AIM2, NLRC4, NLRP3 and NLRP1 [44, 45]. When activated, all these inflammasomes direct the cleavage of pro-IL-1β and pro-IL-18 to their mature forms, IL-1β and IL-18. Similarly, to cellular death, the inflammasomes are also capable of reaching the same outcome; that is, the production of IL-1β and IL-18, via different signalling pathways. Some studies have reported overactivation of the NLRP3 inflammasomes in patients with CF, leading to excessive production of IL-1β and IL-18 [28, 46–48]; however, these various mechanisms are not the only redundant cellular pathways in mammalian cells. Many other mechanisms serve to induce alternative compensatory signalling pathways involving inflammation, metabolism, endoplasmic reticulum (ER) stress, ion transport and phagocytosis. For example, in CF, the lack of complete CFTR functionality leads to reduced transport of Cl^−^ into the extracellular space [9, 49]. This ionic imbalance will lead to upregulation and overactivity of the epithelial sodium channel (ENaC), resulting in sodium (Na^+^) influx into the cells, followed by water; thereby, causing dehydration of the airway surface liquid (ASL) layer in the lumen of the lungs [9, 28, 50, 51]. The mechanisms described above are not the only ones affected in CF; therefore, the mechanistic signalling pathways altered in innate immune cells with CFTR mutations will be analysed in the following sections.

## Innate immune cells with CFTR mutations

### Airway epithelial cells

Airway epithelial cells (AECs) are the initial checkpoints in the defence against pathogens and other inhaled particulates, and these cells are crucial to the regulation of both innate and adaptive immune responses to these challenges [52, 53]. AECs are tailored with innate immune cell machinery such that they are able to detect pathogen-associated molecular pattern molecules (PAMPs) via a broad range of pattern recognition receptors (PRRs) [53–55]. The activation of PRRs (TLRs, NLRs, CLRs and RLRs) triggers intracellular signalling cascades that initiate pro-inflammatory, cytokine and chemokine release and antimicrobial responses [54–57]. In CFTR-deficient AECs, innate immune responses are intrinsically defective, resulting in altered pathogen interactions and immune cell communication. Several studies have described intrinsic upregulation of signalling pathways associated with pro-inflammatory cytokine transcription in CF epithelial cells [58–60]. Additionally, when CFTR-deficient AECs are exposed to *Pseudomonas aeruginosa,* there is activation of nuclear factor-κB (NF-κB), which drives the expression of IL-8, a potent neutrophil chemoattractant [61, 62]. Moreover, a recent report showed that elevated intracellular Cl^−^, resulting from defective CFTR ion transport, triggers pro-inflammatory cytokine secretion in CF epithelial cells through phosphorylation of the Cl^−^ sensitive serum- and glucocorticoid-inducible protein kinase 1 (SGK1), with subsequent activation of the NF-κB pathway [63]. *P. aeruginosa* lipopolysaccharide (LPS) stimulation also increased intracellular Cl^−^ levels, and triggered NF-κB activity in an SGK1-dependent manner, suggesting that alterations in intracellular Cl^−^ may be a cause of both infection-dependent and -independent inflammatory responses [63]. Another study suggested that Cl^−^ concentrations above 75 mM, in the IB3-1 CF epithelial cell line, can modulate IL-1β maturation, suggesting that Cl^−^ itself can enhance inflammatory signalling in these cells [64]. Although, these studies demonstrate that the CFTR may be intrinsically proinflammatory, other reports have shown a high variability in AEC inflammatory responses [39, 65], with conflicting evidence found in vitro and in vivo studies [66–68]. It is important to mention that conflicting evidence may be due to differences in cell lines or the culturing conditions, and that all studies need to be analysed carefully.

**Fig. 1:**
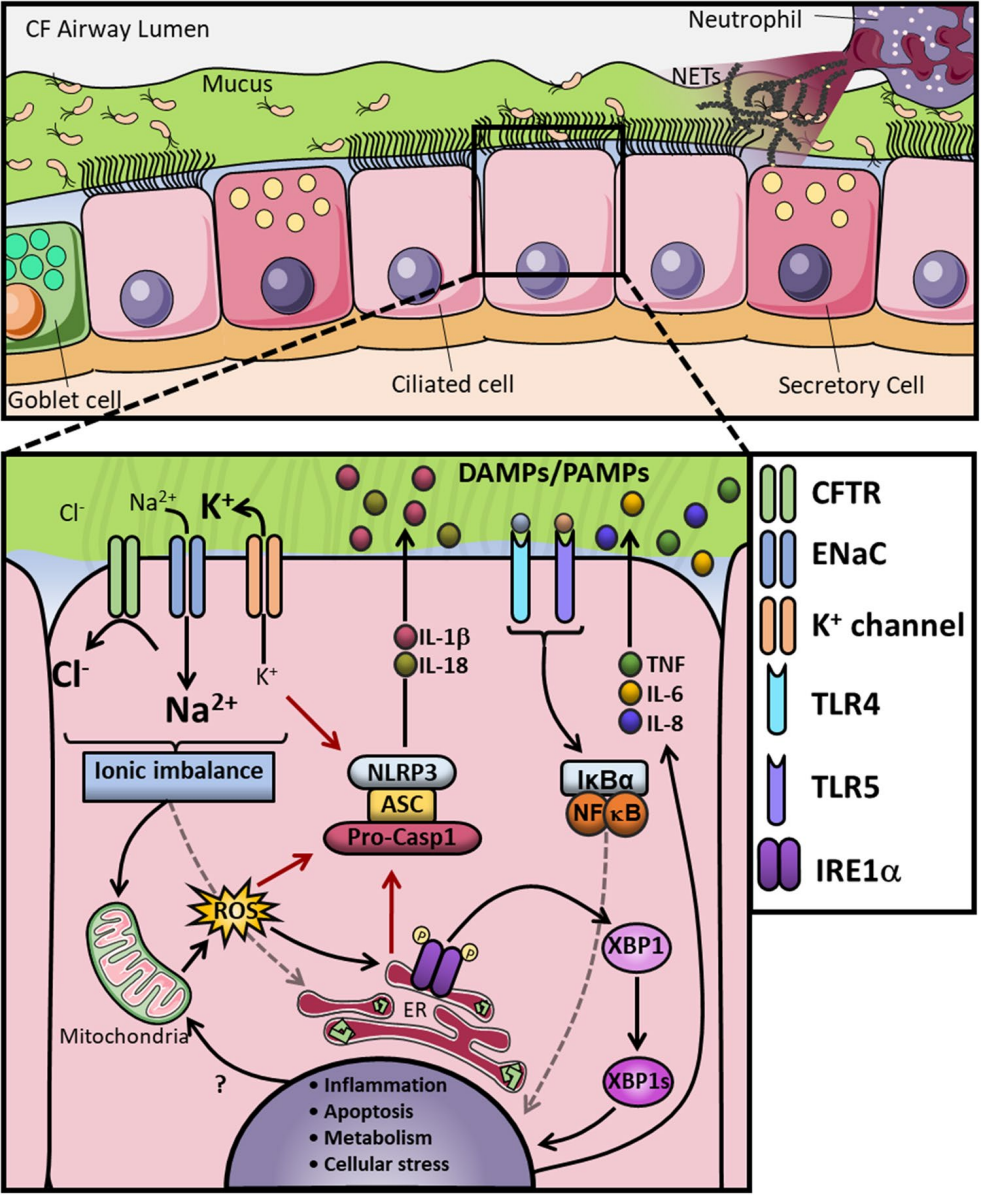
CF airway and altered AECs mechanisms. **a** In this panel, a cross-section of the CF airways is represented, which showing the airway lumen on top and different epithelial cells on the bottom. In CF, the lack of CFTR function leads to increased Na^+^ influx by ENaC, followed by water absorption leading to dehydration of the periciliary layer (PCL), with accumulation of a thick, dense mucus in the apical surface and persistent colonisation by opportunistic pathogens. The chronic inflammatory microenvironment in the lung facilitates neutrophilic infiltration, with subsequent release of excessive amounts of neutrophil extracellular traps (NETs) upon activation. **b** In CF AECs the CFTR malfunction decompensates the intracellular ionic balance, leading to overactivity of ENaC and increased Na^+^ influx and K^+^ efflux, as a consequence. This exaggerated K^+^ efflux, combined with increased ER stress and reactive oxygen species (ROS) production, activates the NLRP3 inflammasome and further increases IL-1β and IL-18 secretion. The ionic imbalance is also associated with increased ER stress, ROS and metabolic turnover. The misfolded CFTR, combined with the ionic imbalance, causes IRE1α activation with the generation of the spliced form of XBP1 (XBP1s), which, in turn, activates a number of UPR-related genes inducing inflammation. The overstimulation of both surface and intracellular receptors, through DMAPs and PAMPs, combined with all the other dysfunctional signalling pathways, causes an exacerbated inflammatory response with increased production of TNF, IL-6 and IL-8

While the central role of the CFTR channel is to balance the flux of Cl^−^ and bicarbonate ions [69] to maintain a healthy ASL, several groups have proposed that the CFTR also influences the activity of various other ion channels, transporters and receptors [70], which may all contribute to the altered innate immune response seen in CF epithelial cells. Most notably, the CFTR has been shown to exert an inhibitory effect on ENaC [71, 72]. Human studies have indicated that variants in genes encoding ENaC chains can cause functional abnormalities, which result in CF-like symptoms, and that rare mutations causing reduced ENaC activity can slow disease progression in patients homozygous for CFTR ΔF508 mutation [73, 74]. Furthermore, ENaC β-chain expression was found to be upregulated in human bronchial epithelial cells (HBECs) with CF-associated mutations (IB3-1 and CuFi-1) and CF monocytes (with ΔF508/ΔF508 mutations), leading to increased Na^+^ influx and K^+^ efflux. This ionic imbalance acts as a driving force for activation of the NLRP3-inflammasome [75–78]. In this study, an exaggerated pro-inflammatory response is seen in CF cells when challenged with NLRP3-inflammasome activators LPS and ATP, leading to increased IL-18 secretion in CF HBECs, and IL-1β and IL-18 in CF monocytes (Fig. 1) [28]. This exaggerated inflammatory response was reduced when cells were pre-treated with small molecule inhibitors of NLRP3 and ENaC; thereby, uncovering a molecular link between enhanced ENaC-dependent Na^+^ influx, K^+^ efflux and NLRP3-inflammatory cytokine production [28].

Under normal conditions, the transport of Cl^−^ ions is coupled to an increase in K^+^ conductance, in order to maintain the driving force for anion movement across the membrane [79]. The KCa3.1 calcium-activated potassium channel, encoded by the *Kcnn4* gene, is essential in calcium-activated intestinal anion secretion [80, 81], and physically interacts with CFTR in the apical membrane of airway epithelial cells [82]. KCa3.1 and CFTR co-localise at the plasma membrane with aggregation of KCa3.1 channels, leading to an enhanced interaction with CFTR channels, following an increase in intracellular calcium (Ca^2+^) concentration [79]. The *Kcnn4* gene has been suggested as a putative modifier of CF severity in humans [83], and it was thought that increased activity of KCa3.1 might aid in counterbalancing the failure of anion and fluid secretion in intestinal epithelial cells. In a recent study, a double mutant mouse (Cftr^ΔF508/ΔF508^ and Kcnn4^−/−^) showed improved survival without alteration in the intestinal secretory function, when compared with the Cftr^ΔF508/ΔF508^ mouse [83]. Interestingly, it was found that inhibiting the KCa3.1 channel, in the Cftr^ΔF508/ΔF508^ mice, reduced lethality and decreased the level of circulating TNF [83]. It would be interesting to explore whether NLRP3-inflammasome activation, IL-1β and IL-18 are also reduced with KCa3.1 channel inhibition. Expanding on this, Phillip et al. explored Th2 responses and found that silencing the STAT6 regulator of the Th2-driven immune response significantly reduced lethality in the CF animals [84]. This supports evidence from studies in macrophages, whereby an imbalance in polarisation towards the M1 pro-inflammatory phenotype suggests a defect in the STAT6 pathways, and interestingly, inhibition of the KCa3.1 channels reduces M1 polarisation [85]; however, the macrophage imbalance is likely to be a combination of several factors as it will be discussed in the macrophage section [86]

Another channel that is altered in CF is the Ca^2+^ release-activated Ca^2+^ channel, ORAI1. Balghi and colleagues found a twofold elevation in the expression of ORAI1 in the cell membrane of Cftr-deficient ACEs [87]. They also reported a twofold increase in total intracellular Ca^2+^ and iCRAC current and a corresponding twofold increase in IL-8 secretion. This increase in Ca^2+^ is likely to trigger the conductance of the KCa3.1 channel and enhance inflammatory signalling, as described above. It has been suggested that the KCa3.1 channel is controlled by Ca^2+^ microdomains, and that KCa3.1 and Orai1 form part of a macromolecular complex organised by PDZ-containing scaffolding proteins [88].

TMEM16A (anoctamin 1) is a non-CFTR Cl^−^ channel which is also activated by Ca^2+^. A recent study by Benedetto et al. found that TMEM16A is not only essential for Ca^2+^ activated chloride currents in both mouse intestine and airways, but it is also essential for the correct activation and membrane expression of CFTR, suggesting an overlap in CFTR- and Ca^2+^- dependent chloride transport [89]. Comparable to KCa3.1 and Orai1, TMEM16A also interacts with CFTR via PDZ-domain proteins [89]. Considering the increased levels of Ca^2+^ in CF AECs and subsequent increased expression of ORAI1, ensuing upregulation of TMEM16A could also be expected; nevertheless, a study on cultured HBECs reported no differences in the expression of TMEM16A between CF and non-CF cells [90]. When CF AECs are exposed to pyocyanin (a major virulence factor of *P. aeruginosa*), the expression of TMEM16A and mucin 5AC is increased, causing mucus hypersecretion [90]. While under normal conditions, TMEM16A supports Cl^−^ conductance and fluid secretion in ciliated AECs, during inflammation TMEM16A expression is upregulated, primarily in mucus-producing goblet cells, leading to excessive mucus secretion, with little expression induced in the ciliated epithelial cells that express CFTR [89]. Kunzelmann et al. suggest that, in the absence of bacterial infections, intrinsic inflammation could be caused by delocalisation/dysfunction of CFTR, followed by upregulation of TMEM16A (possibly as a result of increased intracellular Ca^2+^), particularly in mucus-secreting cells thereby contributing to CF pathogenesis [91]. Another study reported that the overexpression and induction of TMEM16A attenuate the production of pro-inflammatory cytokines, IL-6, IL-8, CXCL1-3 and CCL2 in CF HBECs in air–liquid culture; however, the mechanism of action remains elusive [92]. It seems that augmenting mucus secretion through activation of TMEM16A would over-ride this phenomenon resulting in increased pro-inflammatory cytokine production, as a consequence of increased mucus build-up and bacterial colonisation. Potentially, a more direct approach to TMEM16A activation could be to specifically target ciliated AECs and activate TMEM16A allosterically without triggering Ca^2+^ signalling, which would induce pro-inflammatory cytokine secretion [93]. Alongside CFTR-mediated ion channel disturbance in CF, a number of studies have reported dysregulated or excessive apoptosis in CF epithelial cells following external stimuli [61, 94]; a recent report shows that, under basal conditions, Fas expression was increased in epithelial cell lines leading to increased activation of caspases-3 and -8 and subsequent apoptosis. Interestingly, treatment of primary non-CF bronchial epithelial cells with a CFTR inhibitor resulted in increased Fas expression, suggesting a link between CFTR function and Fas expression [95].

The CFTR receptor itself also has a direct role in the clearance of pathogens which enter the lung. When exposed to infection, the CFTR protein acts as a receptor for *P. aeruginosa* in AECs, and it is thought to be involved in uptake and clearance of this pathogen [96, 97]. *P. aeruginosa* also induces gene expression via activation of TLRs. CF AECs exposed to this pathogen release excessive amounts of pro-inflammatory cytokines, mainly mediated by TLR4/LPS and by TLR5/flagellin interactions [98, 99]. Dysregulation of intracellular TLR4 trafficking has also been noted in human CF AECs as compared to non-CF controls [100–102]. Epithelial cells lacking CFTR also have impaired uptake and breakdown of conidia, which is released from *Aspergillus fumigatus* spores [103]. The soluble pattern recognition receptor, pentraxin 3 (PTX3), is secreted by a variety of immune cells including AEC [104], in response to conidia and is involved in the recognition, uptake and killing of conidia [105]. Furthermore, neutrophil elastases and *A. fumigatus* proteases were found to be responsible for the degradation of the conidial recognition site at the N-terminus of PTX3, contributing to inefficient fungal clearance in CF [106, 107]. Recent human studies, using the CFTR modulator Ivacaftor, found a decreased occurrence of *A. fumigatus* in sputum cultures in CF patients with a G551D mutation; however, the mechanism remains undetermined [108]. Collectively, these studies suggest that loss of functional CFTR and the compound effects of ionic imbalance could prime the innate immune system in advance of infection, leading to heightened inflammatory responses and pro-apoptotic priming when an infection is present (Fig. 1).

## Neutrophils

Neutrophils are commonly considered to be the first line of defence against infection and whilst they are the most abundant circulating leukocyte in human blood, constituting about 60% of white blood cells, the number of neutrophils present in the pulmonary capillaries is increased in CF [109, 110]. Upon interaction with a broad range of pathogens, pro-inflammatory cytokines or other inflammatory signals, neutrophils become activated and move towards the site of inflammation, where they mobilise to clear the invading organism through phagocytosis [111–113], with the release of neutrophil extracellular traps (NETs) [114, 115], as well as cytokines and chemokines [116, 117]. Neutrophils are key inflammatory cells in the immune system’s arsenal, and the dysregulation of approximately 90 of their genes related to the production of cytokines, chemokines, interleukin receptors, colony-stimulating factors and intracellular signalling molecules dramatically contributes to the inflammatory phenotype found in CF [118]. This dysregulation may also lead to an increased risk of infection taking place in the lungs by *P. aeruginosa* [119, 120], *Staphylococcus aureus* [121] and *Burkholderia cepacia* complex [122].

The CFTR has been shown to be expressed on the cellular surface of neutrophils, indicating that their dysregulation is primarily caused by the absence of a functioning CFTR protein, as opposed to just being a secondary effect of mutated CFTR in epithelial cells [123]. Typically, the CFTR is recruited to phagosomes in neutrophils, assisting in the killing of phagocytosed pathogens by moving Cl^−^ ions into the phagolysosome to produce hypochlorous acid (HOCl) [124, 125]; however, in neutrophils with CF mutations, the dysfunctional CFTR compromises the ability of neutrophils to kill pathogens due to defective HOCl production in these compartments [124, 126, 127]. Although another study was unable to find impaired phagocytosis of *P. aeruginosa* in neutrophils with CF mutations, they did find that phagocytosis is impaired in monocytes from patients with CF [128]. The treatment of CF patients, carrying at least one copy of the G551D mutation, with Ivacaftor leads to reduced migration and activation of neutrophils [129], as well as improved bacterial clearance in this cohort of patients [127]. Another potential therapeutic target for CF was proposed to be histone deacetylase 6 (HDAC6), as its depletion, in a mouse model, reduced the recruitment of neutrophils to the lungs, followed by an improved response to infection and an increased rate of bacterial clearance and reduced weight loss [130].

Neutrophils have the ability to expel both their nuclear and mitochondrial DNA coated with antimicrobial granular proteins, such as neutrophil elastase (NE) and myeloperoxidase (MPO), into the extracellular space in net-like structures called NETs, which are used to enmesh pathogenic microorganisms, thereby aiding in their clearance [131]. Before the discovery of NETosis (controlled neutrophil cell death with the release of NETs), necrosis was generally considered to be the primary source of neutrophil DNA in the lungs of patients with CF; however, this was subsequently revealed not to be the case, as NETosis was shown to be responsible for the release of myeloperoxidase (MPO), heparin-binding protein (HBP), DNA and NE in CF sputum and bronchoalveolar lavage fluid (BALF) [132–135]. As the high concentration of inflammatory markers and these proteins, both in the CF sputum and BALF, correlates with decreasing lung function, NETosis plays an essential role in the pathogenesis of CF [132, 134, 136–146]. The raised levels of NE and cathepsin G have been suggested to be responsible for increased levels of peptides and amino acids in sputum samples, whilst the raised concentrations of these peptides and amino acids correlate with increased frequency of *P. aeruginosa* infection in the lungs of patients with CF [147]. The dysregulation of NETosis plays a prominent role in CF as well as other autoinflammatory diseases, as the DNA from these NETs exacerbates the dehydration of mucus, resulting in further airway clogging, thereby producing, as a consequence, an environment which is prone to bacterial infections [131, 148]. Unrestricted NE activity, in 3-month-old infants with CF, has been associated with the development of bronchiectasis, adding to the evidence that respiratory infections in CF lead to neutrophilic infiltration, inflammation and ultimately to a declining respiratory function [149, 150]; nevertheless, a recent study found no association between inflammation, abnormal physiology and structural changes in one-year-old infants, contradicting previous reports [151].

The recent observation of autoantibodies against components of NETs, in patients with CF, has been correlated with declining lung function and provides an insight into the development of autoimmunity in CF [152, 153]. Impaired degranulation, due to decreased Rab27a activity and delayed neutrophil apoptosis, leads to an excessive NET formation in the lungs of patients with CF, and these defects are reversed by Ivacaftor therapy in patients with G551D mutations [154, 155]. Some of the current therapies, such as the use of DNase, to help clear the viscous mucus in the lungs of patients with CF, may exacerbate NE activity, while the use of protease inhibitors has been shown not to affect CF sputum [156]. Recently the exhaled breath condensate (EBC) test was investigated to establish whether there was a link between inflammatory markers in the EBC and clinical outcome. NE was the only inflammatory marker found to be raised in EBC; conversely, this did not correlate with clinical outcome in these patients [157]. As, it has been reported that NE directly targets the CFTR interfering with its functionality [158], studies have aimed to inhibit NE and establish why previous NE inhibition studies have had mixed results [159, 160]. In CF, micro RNA (miRNA) miR-636 was found be upregulated in neutrophils, and has been suggested to play an essential role in the chronic inflammation seen in CF by decreasing the expression of IL1R1 and IKKβ proteins, as well as increasing the expression of RANK [161]. Increased expression of the chemokine receptor, CXCR4, was found on neutrophils with CF mutations [162]; moreover, these raised levels in CXCR4 were associated with chronic fungal infection by *Aspergillus fumigatus* in patients with CF [163]. The cleavage of another chemokine receptor, CXCR1, has also been linked to impaired bacterial clearance in CF [164]. Other pro-inflammatory signals, such as ROS [165] and colony-stimulating factors [166], are significantly raised in CF; likewise, levels of the bioactive fragment of collagen called proline-glycine-proline (PGP) which is generated by the activity of both prolylendopeptidase and matrix metalloproteinase-9 (MMP9), are also raised [167]. As PGP acts as a stimulant of neutrophil migration into tissues, as well as inducing epithelial remodelling, it has been suggested that the raised levels of PGP found in the lungs of children with CF may exacerbate the inflammatory phenotype found in these patients [167]. The implications of the CFTR in neutrophil activity are more than clear and perhaps these new insights can help to achieve better understanding of these myeloid cells in the pathogenesis of CF.

## Monocytes

Monocytes are central circulating white blood cells originating from the bone marrow with the potential to be differentiated into macrophages and dendritic cells, which are professional antigen-presenting cells (APC) involved in the interaction with the adaptive immune system. The CFTR plays a vital role in myeloid cells, and it has been shown that in conditional KO mice models, where only myeloid-derived cells lack CFTR expression, the absence of the CFTR was directly involved in cell function [168]. Under normal conditions, these myeloid CFTR KO mice did not show any visible phenotypical alterations as compared to their WT counterparts; interestingly, when the lungs of these mice were exposed to bacterial pathogens, the rodents displayed significantly higher amounts of inflammatory cytokines and a decreased survival rate as compared to WT mice [168]. Although this study did not directly associate monocytes with the atypical response to bacteria in these mice, other studies have shown the importance of the CFTR in the regulation of monocyte function [24, 25, 27, 169]. The expression of the surface markers CD14 and HLA-DR was shown to be downregulated in monocytes from children with CF, alongside with deficient phagocytosis [170]. Later reports showed no differences in the monocyte classical (CD14^++^ CD16^−^), intermediate (CD14^+^ CD16^+^), or non-classical (CD14^+^ CD16^++^) subpopulations [25, 171]; however, expression of M-CSF, TLR4, IL-4Rα, IL-13Rα1, TIMP-1 and Cox-2, were shown to be upregulated in monocytes from CF patients, demonstrating that CFTR mutations intrinsically affect these myeloid cells [25]. TLR4, in particular, has been consistently reported to be upregulated in both monocytes and macrophages from patients with CF and these increased levels of TLR4 were not related to pulmonary infections [24, 25, 102]. This persistent TLR4 upregulation might be linked to the exaggerated inflammatory response seen in patients with CF. Freshly isolated monocytes from patients with CF did not show any differences in the surface marker expression of CD68 and CD80, but significantly higher expression of CD163 and CD206 was observed when compared with HC monocytes [172]; however, the monocytes of two different patient cohorts, on Ivacaftor and Ivacaftor/Lumacaftor, showed normalisation of the increased levels of CD163 and CD206, which were comparable to the levels of HC monocytes [172]. The surface markers CD68 and CD80 are associated with a pro-inflammatory phenotype, while the CD163 and CD206 are linked to an anti-inflammatory phenotype, suggesting that there is a persistent and non-resolving inflammatory response in CF [172]. These studies provide more evidence that monocytes with CF mutations show phenotypic alternations that can be improved with the administration of CFTR modulators. In a different study, IL-8 was shown to be abnormally increased in CF monocytes after LPS challenge; however, these higher levels of IL-8 were not associated with TLR4 overexpression but, instead, with an increase in the MAPK signalling pathway [169]. In another study, it was shown that the monocytes from patients with CF, with at least one copy of the G551D mutation, treated for seven days with Ivacaftor displayed a reduction in IFNγ induced related inflammatory proteins [173]. Similarly, Velard et al. found that the percentage of double positive monocytes, RANK^+^ and M-CSFR^+^, was strongly increased (~ 91%) in CF patients bearing at least one G551D copy when compared with HC [174]; interestingly, this increased percentage in RANK^+^ and M-CSFR^+^ monocytes, was significantly decreased after 9 and 12 months of treatment with Ivacaftor [174]. It is of great interest to investigate how this increased expression in M-CSFR is involved in the differentiation process of monocytes towards macrophages and dendritic cells in patients with CF.

Monocytes with CFTR mutations show an exaggerated inflammatory phenotype when stimulated with LPS and ATP, and this activation state was NLRP3 dependent [28]. Monocytes from patients with CF showed increased production of IL-1β and IL-18, which was associated with higher activity of caspase-1 and raised extracellular ASCs [28]; interestingly, inhibition of ENaC decreased the exacerbated secretion IL-1β and IL-18 only in monocytes with CF mutations, while this inhibitory effect was not seen in monocytes from patients with other inflammatory conditions [28]. Furthermore, the increased levels of IL-1β and IL-18 were also detected in the sera of patients with CF [28], indicating an essential role of these two cytokines in the pathogenesis of this disease, further supporting the autoinflammatory phenotype seen in patients with CF [31, 175, 176]. Moreover, the CFTR modulators Ivacaftor/Lumacaftor and Ivacaftor/Tezacaftor showed a powerful anti-inflammatory effect in patients with CF, reducing the levels of IL-18, and both IL-1β and IL-18, correspondingly, in monocytes and serum of patients with CF [177]. An altered X-linked miRNA profile has been shown in CD14^+^ monocytes from patients with CF [178]. In this study, the authors found that several X-linked miRNAs were significantly upregulated in CF monocytes, with miR-224-5p being the most prominent; furthermore, SMAD family member 4 (SMAD4), a validated target of miR-224-5p was found to be downregulated in the CF monocytes [178]. The full implications of miRNAs in monocytes with CF mutations are still unknown, but these inhibitory miRNAs are indeed implicated in the pathogenesis of CF [179]. In a different study, transcriptomic RNA sequencing (RNAseq) was carried out in whole blood from patients with CF, and 491 genes were found to be differentially expressed as compared to non-CF controls, with further validation of the most overexpressed genes, MMP9 and SOCS3 by qPCR [180]. It has been shown that monocytes from patients with CF have increased expression of MMP9, associated with increased intracellular Ca^2+^ when compared with non-CF monocytes [180, 181]; remarkably, monocytes with from CF patients treated with Ivacaftor/Lumacaftor showed reduced intracellular Ca^2+^ levels [180]. Finally, a recent study showed that overexpression of PD-L1 in monocytes with CF mutations, was associated with *P. aeruginosa* infections in patients with CF [182]; furthermore, not only PD-L1 was increased in the CF monocytes, but also the levels of sPD-L1 were increased in the plasma of these patients, as well as PD-1 in both CD4^+^ and CD8^+^ T cells [182]. Clearly, several distinct mechanistic pathways are dysregulated in monocytes with CF mutations, and a better understanding of all these mechanisms is needed.

## Macrophages

**Fig. 2:**
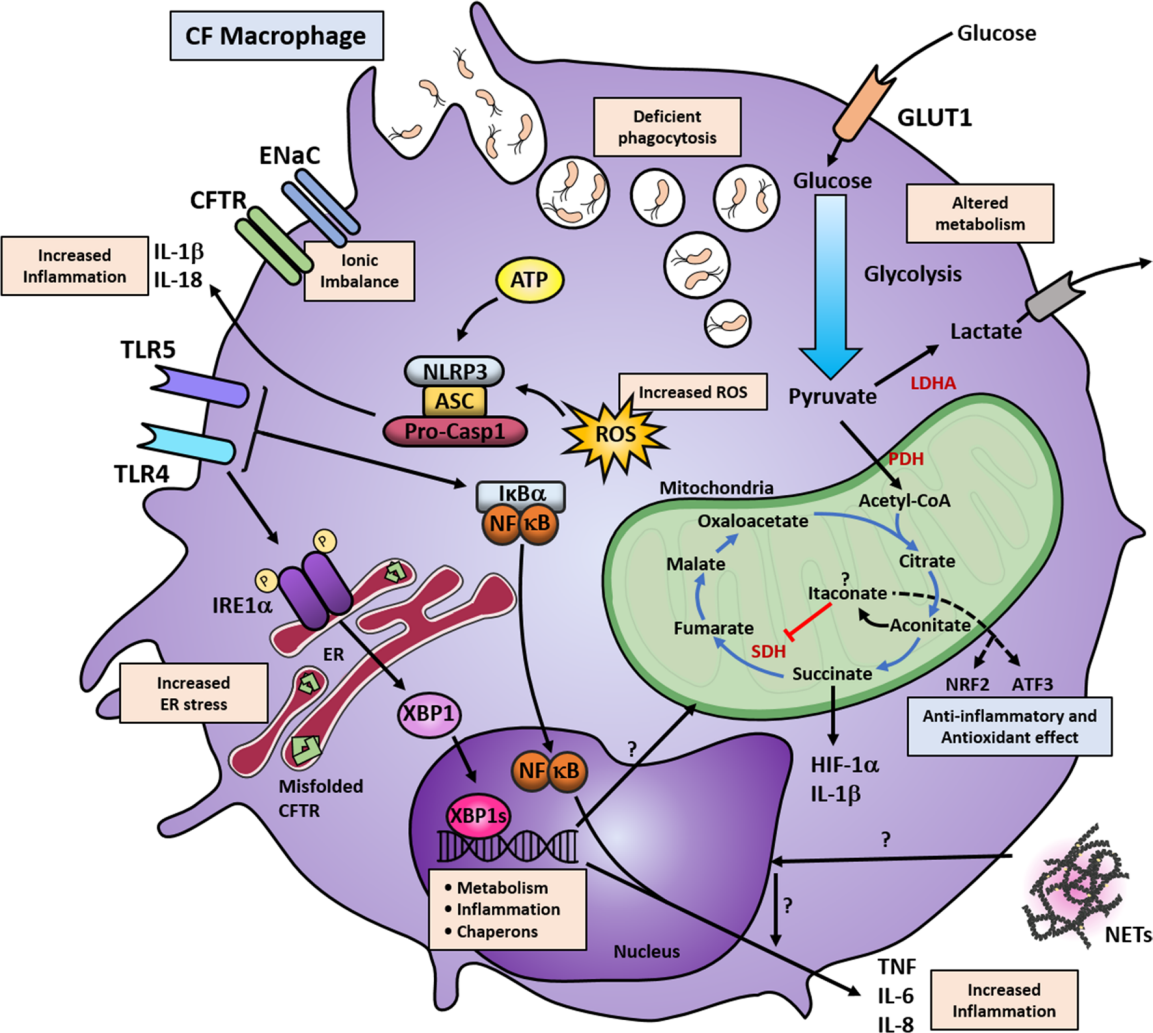
Altered signalling pathways in CF macrophages. Macrophages with CFTR mutations show alterations in multiple cellular pathways. The mutated CFTR causes ionic imbalance, with accumulation of misfolded protein in the case of the ∆F508 mutations and primes these myeloid cells towards an altered immune response or chronically activating other signalling pathways. CFTR malfunction primes the overactivation of ENaC, leading to increased Na^+^ influx, which is then compensated by K^+^ efflux. The increased K^+^ efflux, combined with increased ROS and ATP production, activates the NLRP3 inflammasome with further increased IL-1β and IL-18 secretion. CF macrophages have raised levels of TLR4 expression, and the resultant overactivation of NF-κB leads to increased TNF and IL-6 production. Induction TNF and IL-8 may also occur through NETs by an unknown mechanism. Similarly, chronic TLR4 activation, possibly due to the persistent bacterial colonisation in the lungs, leads to the overactivation of IRE1α; thereby, triggering XBP1s. This production of XBP1s induces transcriptional activation of several UPR responsive genes involving metabolism, inflammation and protein folding. XBP1s overexpression induces a low-grade chronic induction of IL-6 and TNF, which exacerbates the inflammatory response when combined with other signalling pathways. XBP1s also regulate metabolic pathways and, in CF macrophages, the increased metabolic state can be reduced by IRE1α inhibition. Macrophages with CFTR mutations also show increased glycolytic flux and mitochondrial respiration. It is known that in M1 macrophages the Krebs cycle favours the accumulation of succinate and citrate. Succinate accumulation leads to stabilisation of HIF-1α, which can induce IL-1β production and activation of glycolytic genes. It may be possible that in CF macrophages, this axis is favouring a proinflammatory response and increased glycolytic function. Alternatively, citrate is converted into aconitate, facilitating the synthesis of itaconate, which is a potent anti-inflammatory metabolite; however, the role of itaconate in CF is unknown. CF macrophages also display deficient bacterial killing with intracellular accumulation of phagocytic vesicles. Altogether, these mechanisms influence the altered innate response elicited by macrophages. *LDHA* lactate dehydrogenase A, *GLUT* glucose transporter, *SDH* succinate dehydrogenase, *PDH* pyruvate dehydrogenase, *ER* endoplasmic reticulum, *HIF-1α* hypoxia inducible factor 1 subunit alpha, *NRF2* nuclear factor erythroid-2-related factor 2, *ATF3* activating transcription factor 3, *ROS* reactive oxygen species

As already described, inflammation is a common complication in CF. Although epithelial cells, neutrophils and monocytes play an essential role in the pathogenies of CF, macrophages are largely responsible for the initiation and resolution of the inflammatory response [183–185]. During inflammation, monocytes are recruited to the affected site and these myeloid cells can then be differentiated and subsequently polarised into classically activated macrophages or alternatively activated macrophages, better known as pro-inflammatory (M1) and anti-inflammatory (M2), respectively [186]. Although distinct molecular signalling pathways in monocyte-derived macrophages (MDMs) are known to be affected by CFTR mutations [26, 27, 154, 172, 187], it is important to mention that other types of macrophages exist, known as tissue-resident macrophages, which are also affected by these types of mutations [30, 188, 189]. The proportion of macrophages is typically elevated in the airways, both in patients with CF and mice with CFTR mutations [86, 190, 191]. Furthermore, M1 and M2 macrophages are reported to be increased in the lung and peritoneum of mice with CFTR mutations [86]. Deficient polarisation of M2 MDMs has been reported in human macrophages, with significantly lower production of IL-10 and absence of expression of IL-13Rα1, while no difference was observed in the proportion of M1 MDMs [26, 27]. While no proportional differences were found in M1 MDMs with CFTR mutations, there is substantial evidence to show that these cells consistently secrete excessive amounts of pro-inflammatory cytokines, including TNF, IL-1β, IL-6, IL-8 and IL-12, under basal conditions, and also after stimulation (**Fig. ****2**) [27, 154, 172, 189, 192]. Furthermore, the upregulation of the transcription factor XBP1s has been reported in lung tissue-resident macrophages and M1 MDM from patients with CF, in association with excessive production of IL-6 and TNF [27, 30, 189]. Moreover, a hypermetabolic state has been reported in M1 MDMs from patients with CF, showing higher glycolytic and mitochondrial activity associated with increased activity of the IRE1α-XBP1 signalling pathway [27]. Interestingly, it has been previously hypothesised that the chronically raised levels of pro-inflammatory cytokines might result in metabolic disturbances during the pathogenesis of CF [193, 194]. Certainly, this is the case in other immune conditions, where the excessive levels of pro-inflammatory cytokines, as well as other intrinsic cellular abnormalities, contribute to the induction of cellular stress, which is directly associated with disruption of cellular metabolism [195–198]. It would be interesting to investigate the effects of the increased levels of pro-inflammatory cytokines, present in patients with CF, concerning glycolytic and mitochondrial metabolism.

Exaggerated inflammatory and metabolic responses are not the only altered pathways in macrophages harbouring CFTR mutations. It has been reported that such macrophages display increased apoptosis and deficient bacterial killing, associated with decreased phagocytic capacity (**Fig. ****2**) [172, 199, 200]. MDMs with CFTR mutations showed a 40% reduction in phagocytosis, which could be recovered under the administration of Ivacaftor, but not Ivacaftor/Lumacaftor; this recovery was associated with a significant increase in the killing of the opportunistic pathogen *P. aeruginosa*, by CF MDMs treated with Ivacaftor [172]. The deficient phagocytic response towards *P. aeruginosa* by CF macrophages, might be due to the lower expression in TLR5, as this is the preferred receptor used by macrophages to detect this pathogen [201]. As discussed already, NETs are increased in the lungs of patients with CF and, interestingly, the production of NETs in response to *P. aeruginosa* infections is influenced by macrophage migration-inhibitory factor (MIF), via induction of mitogen-activated protein kinase [135]. In a different study, Gray et al. reported that NETs could also induce the production of TNF and IL-8 in human MDMs, with significantly increased levels of these two cytokines in CF MDMs (Fig. 2) [154].

Macrophages are undoubtedly dysfunctional or overactive in patients with CF, showing excessive amounts of pro-inflammatory cytokine production, altered polarisation ratios with reduced numbers of M2 MDMs, increased metabolic rates, as well as deficient phagocytosis and killing properties. It would be interesting to elucidate whether these macrophage dysfunctions are due to intrinsic or extrinsic influences, or indeed a combination of both. Recently, itaconate has been described as a potent anti-inflammatory metabolite implicated in metabolic reprogramming during macrophage activation [202–204]. When macrophages become activated, aconitate is converted to itaconate in the Krebs cycle, and it is known that itaconate acts as an anti-inflammatory molecule, similar to IL-10 [202–204]. Further investigation of whether itaconate and other metabolites are dysregulated in macrophages with CFTR mutations is in progress. As the triple-drug combination therapy, Trikafta, has been recently approved in the US, it will be of particular relevance to investigate the effects of this drug combination on macrophages with CFTR mutations. Altogether, it is clear that the CFTR plays a significant role in the regulation of several mechanisms in macrophages, and a dysfunctional CFTR can lead to several cellular abnormities in these phagocytic cells.

## Fibroblasts

Fibroblasts are essential stromal cells that are involved in the regulation of the tissue environment, wound healing, angiogenesis and tissue fibrosis. Recently, several studies have demonstrated that these cells are essential in the regulation of the inflammatory response and that they play a crucial role in the pathogenesis of several immune-related disorders [205, 206]. Although there is limited information about fibroblasts in CF, it is known that the CFTR is expressed in fibroblasts [193, 207, 208]. Early studies have shown that CF fibroblasts display an altered glycolytic metabolism, with increased activity of glycolytic enzymes [193]. Moreover, lung fibroblasts with CFTR mutations are overresponsive when stimulated with LPS, secreting excessive amounts of TNF, IL-1β and IL-6, when compared with WT fibroblasts [208]. All this is in line with the reports showing increased inflammatory markers in the lungs of patients with CF. It would be of great interest to further investigate whether the chronic activation of fibroblasts in CF, due to the long-lasting inflammation in the lungs, is associated with the decline in respiratory function over time. Moreover, it would be of great interest to explore the effect of the CFTR modulators on these stromal cells. Further investigations should be encouraged to foster our understanding of the role of the CFTR in fibroblasts.

## Final remarks

Collectively, the CFTR plays an important role in the regulation of several cellular mechanisms not only in AECs, but also in innate immune cells. CFTR mutations in AECs, neutrophils, monocytes, macrophages and fibroblasts together lead to imbalance of numerous cellular signalling pathways, with negative consequences in several organs. For instance, the accumulation of thick mucus in the lung, leads to colonisation by opportunistic pathogens, such as of *P. aeruginosa*, mainly due to the Cl^−^ and water imbalance [12]. Epithelial cells are then chronically activated, leading to the recruitment of monocytes, macrophages and neutrophils to control the infection (Fig. 1). When these innate immune cells reach the affected site, they take over the inflammatory response leading to an exaggerated and unresolved pro-inflammatory response. Macrophages fail to destroy most of these pathogens, and if the infection is eradicated, these phagocytic cells show a reduced anti-inflammatory reaction, with consequent impaired production of IL-10 (Fig. 2) [27, 172, 199, 209].

Although, a debate still exists as to whether the CFTR is intrinsically proinflammatory or whether it facilitates inflammation indirectly, the fact that inflammation is increased in CF is unquestionable. HBECs obtained from children with CF less than 5 years old, show significantly higher amounts of IL-8 mRNA when compared with control infants, even in the absence of infections [210]. Several studies support the idea that the defective CFTR facilitates inflammation indirectly, and the exaggerated inflammatory response observed in CF may be due to an exacerbated response to pathogens [65, 210–213]. In fact, the exaggerated inflammation seen during the pathogenies of CF must be certainly due to a combination of both, the CFTR exerting an intrinsic proinflammatory effect and also due to the vigorous response to microbial infections. The different multi-organ complications in CF are indeed associated with excessive inflammation, but it remains to be determined how inflammation affects other organs involved in this disease. The recent introduction of new CFTR modulators, such as Trikafta, has proven to be of great benefit to patients with CF; it is of considerable interest to further investigate the effects of these drugs, in different immune cells in the context of CF, and further explore the possibility of testing these drugs in other disorders in order to boost CFTR function.
